# Early functional proprioceptive stimulation in high spinal cord injury: a pilot study

**DOI:** 10.3389/fresc.2025.1490904

**Published:** 2025-02-26

**Authors:** Florence Martinache, Anne-Claire de Crouy, Arnaud Boutin, Jacques Duranteau, Bernard Vigué

**Affiliations:** ^1^ CIAMS, Université Paris-Saclay, Orsay, France; ^2^ Département d’Anesthésie Réanimation, Service de Rééducation Post-Réanimation (SRPR), Hôpital Universitaire de Bicêtre, APHP, Université Paris-Saclay, Le Kremlin-Bicêtre, France

**Keywords:** spinal cord injury, early rehabilitation, intensive care, tendon vibration, proprioceptive stimulation, spasticity, muscle atrophy

## Abstract

**Introduction:**

The first months following a spinal cord injury (SCI) are crucial for promoting recovery. However, patients with high SCIs often require prolonged stays in intensive care units (ICUs), delaying optimal rehabilitation due to limited resources. This study examined the safety, feasibility, and effects on spasticity and muscle atrophy of an early rehabilitation technique using non-invasive sensory stimulation and called functional proprioceptive stimulation (FPS).

**Materials and methods:**

Ten SCI patients were included in this randomized pilot study, with five receiving early FPS and five receiving sham stimulation. Both groups were treated using the Vibramoov, consisting of 12 computer-synchronized vibrators placed on the lower limbs. Treatment sessions lasted 30 min, four times a week, for up to 8 weeks. Spasticity was assessed using the Modified Ashworth Scale, Tardieu Scale, Spinal Cord Assessment Tool for Spastic Reflexes, and a patient self-evaluation with a visual analog scale. Muscle atrophy was evaluated through ultrasonography of rectus femoris thickness and cross-sectional area. The duration of the follow-up period ranged from 6 months to 1 year.

**Results:**

Treatment began early, with a median of 4 days post-injury for both groups. The number of adverse events was similar between groups, with none linked to the intervention. No medium-term effects on spasticity or muscle atrophy could be identified. However, our results show a tendency toward a beneficial short-term effect of FPS on spasticity, observed for all spasticity measurements.

**Discussion:**

This pilot study shows that early FPS is feasible and safe for SCI as early as the intensive care unit stage. We demonstrated that FPS induced a transient relaxation and spasticity reduction that could potentially enhance a rehabilitation session administered shortly after it, but larger studies are needed to determine the medium and long-term effects.

**Clinical Trial Registration:**

ClinicalTrials.gov, identifier (NCT05094752)

## 1 Introduction

Spinal cord injury (SCI) is a dramatic event resulting in long-term disability. In 2019, approximately 0.9 million new cases of SCI were reported worldwide. This represents an estimated global burden of 6.2 million years lived with disability due to this condition (1). Studies on the natural history of SCI have shown that most recovery occurs within the first 3–6 months post-injury, with the most rapid motor recovery rate during the first 3 months (2, 3). This period is, therefore, crucial for these patients. Appropriate care from admission to a specialized intensive care unit (ICU) is essential not only for survival but also to promote recovery. Advances in SCI management, particularly in reducing delays before surgery (4), have facilitated earlier rehabilitation efforts. Hence, early rehabilitation is recommended in existing guidelines (5, 6). While the optimal timing and type of rehabilitation remain unclear (7), a primary goal of early rehabilitation is the prevention of secondary complications (5). Spasticity, which can lead to complications such as pain and contractures, affects 65%–80% of individuals with chronic SCI (8–10). It may also hinder functional recovery (11). However, research on spasticity during the early stages of SCI is limited (12). The risk of problematic spasticity is more frequent at higher levels of SCI (13). At the same time, patients with high-level SCI (above the sixth thoracic vertebra) often require extended ICU stays for critical care, such as respiratory support (14, 15). Unfortunately, neurorehabilitation in these settings is constrained by limitations in practical, human, and material resources (16). In this context, a non-invasive and easy-to-use tool like vibration could be a valuable therapeutic ally. Various types of vibration are used in rehabilitation, including focal and whole-body vibrations (17).

FPS involves focal tendon vibration to stimulate muscle spindles in a way that mimics functional movements and provides movement illusions to the brain when the central nervous system is intact (18–20). This study investigates the potential benefits of early application of functional proprioceptive stimulation (FPS) in SCI patients. We followed patients as soon as their ICU stay to monitor spasticity and muscle wasting. Participants were randomized into two treatment groups to assess early FPS's safety, feasibility, and efficacy in SCI patients.

Focal vibration has been shown to temporarily reduce spasticity in various neurological impairments, including SCI (17, 21). Furthermore, FPS has already demonstrated functional benefits in SCI patients (22, 23) and individuals with other neurological impairments (24). However, these benefits have primarily been studied in chronic and subacute stages of neurological diseases. Hence, one of our objectives is to determine whether early FPS can contribute to spasticity reduction.

The aim of the present study was manifold. We wanted to examine the safety, feasibility, and effects of an early non-invasive FPS rehabilitation procedure on spasticity and muscle atrophy. To test our hypothesis, we conducted a pilot randomized controlled trial on patients with traumatic SCI. First, we hypothesized that early FPS can be safely applied during the initial ICU stay. Second, we hypothesized that this early intervention might reduce spasticity, mitigate muscle wasting, and promote sensorimotor recovery in tetraplegic and high-level paraplegic patients.

## 2 Materials and methods

### 2.1 Participants

Adult patients (18 years old) with acute traumatic SCI admitted to our hospital's ICU were recruited as soon as possible within 10 days of admission. Exclusion criteria included any medical conditions preventing protocol implementation within 10 days post-injury, severe brain injury, and previous spinal cord injury. The treatment began only after obtaining a signed consent from the patient (or their legal representative) and clearance from their attending physician.

### 2.2 Study design and randomization

This study employed a randomized, sham-controlled design. Patients were randomly allocated in a 1:1 ratio to either the intervention or control group. Randomization was stratified to ensure a similar distribution of patients with complete SCI (initial AIS A) and incomplete SCI (initial AIS B, C, D) between groups. Randomization lists were generated using the Randomization.com website.

Data were collected using Goupile, an online editor of eCRF (https://goupile.fr/), and hosted on a certified HDS (Health data host) server.

The study was approved by a regional ethics committee (CPP Ile-deFrance-2, Paris, France). The trial was prospectively registered (ClinicalTrials.gov, NCT05094752).

### 2.3 Vibramoov

FPS was delivered to patients' lower limbs using the Vibramoov (Techno Concept, Manosque, France). This system comprises 12 wireless vibrators (104 × 34 × 39 mm) piloted by a computer via Wi-Fi, each powered by a 5-volt direct current motor. The vibration amplitude ranges from 1 to 2 mm at frequencies between 40 and 100 Hz.

**Figure 1:**
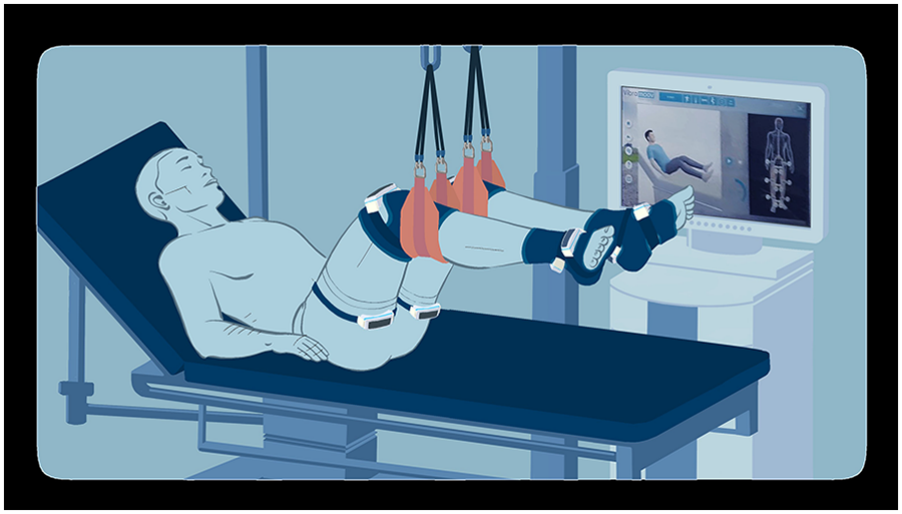
Illustration of the intervention material set on an ICU patient from an observer's point of view.

The vibrators were positioned on the lower limbs as follows: two at the root of the thigh (at the level of the hip flexor tendons above and the hip extensor tendons below), two immediately above the knee (at the level of the quadricipital tendon above and the hamstring tendons below), and two at the ankle and foot (at the level of the tibialis anterior and toe flexor tendons above and the Achilles tendon below), as illustrated in Figure 1. The computer synchronized the vibrators to simulate functional movements.

The system offers three intensity levels, primarily differentiated by dynamics of the frequency slopes during stimulation (intensity 1: 40–65 Hz; intensity 2: 40–80 Hz; intensity 3: 40–100 Hz). These intensities also differ in speed variations, exercise dynamics, and pause duration. During the sessions, an avatar on the screen demonstrates the movements simulated by the vibrators (Figure 1).

The system includes several movement patterns: Flexion/Extension, Walking, Stair Climbing, and Multiactivity, which combines all the movements mentioned above.

### 2.4 Patient's installation

The patients laid on their backs, with the trunk inclined between 30° and 50°. The legs were elevated using straps positioned under the calves and a patient lift, ensuring no contact between the lower limbs and the bed. The hips and knees were maintained in an intermediate flexion position (between 30° and 90°), while the ankles were free (Figure 1).

Vibrators were positioned bilaterally as previously described. To protect the patient's skin from heat generated by the vibrators, a layer of clothing or fabric was placed between the vibrators and the skin.

The system's display screen was positioned on the patient's preferred side (or the only possible side), at a distance allowing clear visibility of the avatar. A cloth was used to obstruct the patient's view of their lower limbs.

### 2.5 Intervention protocol

The treatment was administered throughout each patient's hospital stay, beginning in the intensive care unit (ICU) and continuing through their subsequent care in either the orthopedic or post-ICU rehabilitation unit. The protocol consisted of four 30-min weekly sessions for a maximum of 8 weeks per patient. The intervention group received FPS, while the control group received sham stimulation. Both treatments were delivered using the Vibramoov, with sham stimulation utilizing a modified set of vibrators that excluded two small eccentric weights responsible for generating vibration strength.

For the intervention group, the intensity of vibrations was initially set to 2, and the Flexion/extension movement pattern was selected for the first 4 weeks. This intensity was subsequently increased to 3 with the multiactivity program. All participants received standard care throughout the study period. Trained physiotherapists delivered all the vibration sessions.

### 2.6 Criteria for discontinuation or suspension of treatment

The therapist, in consultation with the patient's physician, was required to terminate the session if any of the following safety criteria were met [Adapted from (25)]:

1. Mean arterial pressure (MAP) ≤60 mmHg
2. Systolic blood pressure (SBP) ≥200 mmHg
3. Heart rate (HR) ≥130 or ≤40 beats per minute
4. Respiratory rate (RR) ≤5 or ≥35 breaths per minute (non-ventilated patients)
5. Pulsed oxygen saturation (SpO2) ≤90%)

It was decided that the treatment would be permanently discontinued if any of these events occurred more than twice consecutively.

### 2.7 Neurological assessment

Neurological function was assessed using the International Standards for Neurological Classification of Spinal Cord Injury (ISNCSCI) in conjunction with the American Spinal Injury Association (ASIA) Impairment Scale (AIS) (26). These evaluations enabled us to determine the AIS grade, Neurological Injury Level (NIL) and Lower Extremity Motor Score (LEMS).

Assessments were conducted at inclusion, ideally within the first week following SCI, and subsequently 1 week after, at 1 month, at 2 months, and, if feasible, between 6 months and 1 year post-injury.

### 2.8 Spasticity assessment

Spasticity of the main muscle groups in the lower limbs (gastrocnemius, soleus, knee flexors, knee extensors, hip adductors) was assessed weekly during the treatment period using the Modified Ashworth Scale (MAS) with a 5-point rating, the Tardieu Scale (TS), and the Spinal Cord Assessment Tool for Spastic Reflexes (SCATS). Patients’ subjective perception of spasticity was also measured using a visual analog scale (VAS). A follow-up assessment of spasticity was conducted between 6 months and 1 year post-injury.

For the gastrocnemius and soleus, spasticity was also measured immediately before and after vibration sessions once weekly during the first 3 weeks of treatment.

The MAS is an ordinal scale with six levels, 0 indicating no spasticity and 5 indicating fixed articulation. The Ashworth scale was initially developed by Ashworth in 1964 (27) and later modified by Bohannon and Smith (28). Its widespread use in literature (29, 30) facilitates comparison with existing studies.

Based on work by Tardieu (31) and Held and Pierrot-Deseilligny (32), the TS was included due to its reported advantage over MAS in some studies (33). It has two main components: the degree of muscle resistance at fast speed (5 levels) and the dynamic component of spasticity (difference in stopping angles at slow and fast speeds) (34), respectively named TS score and TS angle in this study.

Developed by Benz et al. (35), the SCATS was chosen to address the multidimensional nature of spasticity (36–38). It evaluates three typical presentations of spasticity in SCI: clonus of the plantar flexors, flexor spasms, and extensor spasms, each rated from 0 (absent) to 3 (severe).

Finally, a VAS (0–100) was used to capture patients' self-assessment of spasticity-related discomfort.

All spasticity measurements were standardized and performed by a single-trained physiotherapist.

### 2.9 Muscle atrophy assessment

Muscle atrophy was evaluated using ultrasound measurements of the rectus femoris. Assessments were conducted twice weekly for the initial 4 weeks, followed by evaluations at 2 months and between 6 months to 1 year post-injury. Ultrasound measurements of muscle thickness and cross-sectional area provide reliable indicators of muscle wasting and strength loss (39, 40). The protocol for these measurements was adapted from the methodology described by Parry et al. (39).

Images were acquired using two Affiniti 70 ultrasound scanners (Philips, Amsterdam, Netherlands) equipped with a 12–3 MHz linear probe and a Canon Aplio a (Canon Medical Systems, Japan) ultrasound scanner equipped with a 10 MHz linear probe.

For muscle thickness measurements, two anatomical points were identified on the line joining the anterosuperior iliac spine and the upper edge of the patella: one at the midpoint and the other at two-thirds of this line. Three images were taken at each point, and the measurements were averaged to obtain two thickness values.

The cross-sectional area of the rectus femoris was measured at two-thirds of the line joining the anterosuperior iliac spine and the upper edge of the patella. Three images were taken, and the measurements were averaged to obtain the area measurement.

Patient positioning was standardized for each examination: backrest elevated to 30° and knee extended. The lower limb under study was maintained in neutral hip rotation using cushions. A minimum of 10 min elapsed between the last patient mobilization and each ultrasound scan to ensure a stable state away from the muscular effort. During image acquisition, minimal pressure was applied to the probe, and a large quantity of gel was used to ensure satisfactory acoustic contact. Oblique incidence was avoided by obtaining the best possible visualization of the bone-muscle boundary.

**Figure 2:**
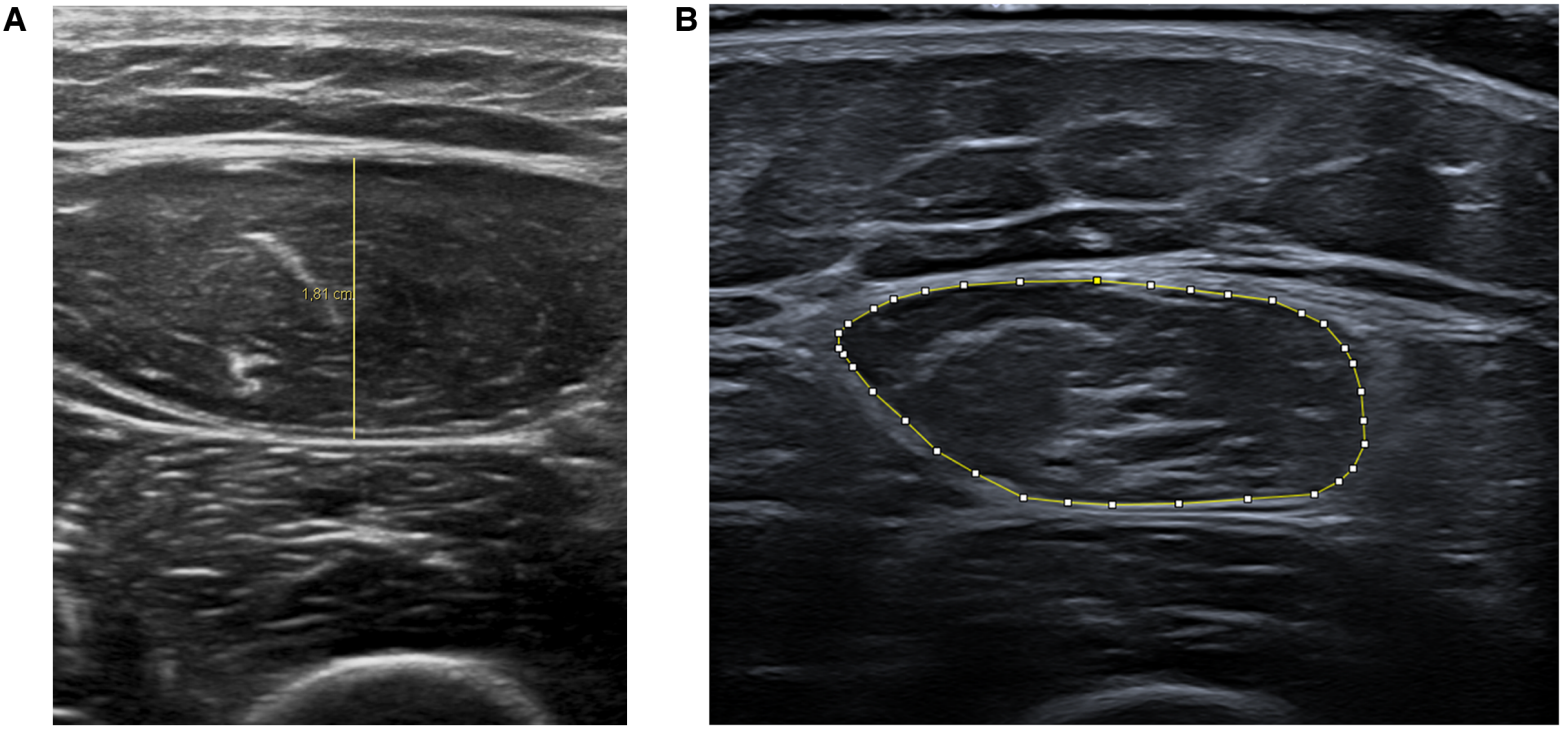
Representative ultrasound images showing rectus femoris muscle thickness **(A)** and cross-sectional area **(B)** measurements.

Muscle thickness measurements (in centimeters) were performed using Philips DICOM Viewer R3.0-SP15 software. Area measurements (in square centimeters) were made using ImageJ software (Figure 2).

### 2.10 Results analyses

Due to the small sample size (*n* = 5 per group), formal statistical tests and *p*-values were not extensively used as they could be potentially misleading. Instead, a descriptive approach was prioritized to highlight potential trends and guide future studies with larger samples. When statistical tests were conducted, non-parametric methods were employed: the Wilcoxon rank-sum test for between-group comparisons and Spearman's correlation coefficients to assess associations between variables. To compare the temporal evolution of variables, we calculated the differences between each time point and computed correlation coefficients for these differences. All statistical analyses were performed using R software (version 4.4.1) (41).

For each participant, weak and strong lower limbs were defined based on the Lower Extremity Motor Score (LEMS) determined by the ISNCSCI. The limb with the lower LEMS was designated as the weak lower limb, while the contralateral limb was considered the strong lower limb. In cases where both limbs had equal LEMS, the limb contralateral to the participant's dominant side was designated as the weak lower limb. Consistency in the designation of weak and strong limbs throughout the follow-up period was verified.

To account for the timing of inclusion, we created bins to compare results at similar intervals post-SCI: first week (1–7 days), second week (8–14 days), third week (15–21 days), fourth week (22–28 days), fifth week (29–5 days), sixth week (36–42 days), seventh week (43–49 days), 2 months (50–71 days), and 6 months to 1 year (180 days and beyond). As shown in Table 1, beyond 35 days, the number of participants is unbalanced between the two groups. Therefore, comparisons beyond this limit were not conducted.

**Table 1:** Number of patients by group and examination interval.

Interval	FPS	Sham stimulation
1–7 days	4	5
8–14 days	4	5
15–21 days	5	5
22–28 days	5	4
29–35 days	4	4
36–42 days	4	1
43–49 days	4	1
2 months	4	1
≥6 months	5	2

Comparisons were made between the two treatment groups, as well as between participants classified as motor complete (AIS A and B) and motor incomplete (AIS C and D). The AIS level used to classify patients as motor complete or incomplete was determined by the one-week examination to account for spinal shock (42).

## 3 Results

### 3.1 Participants

**Figure 3:**
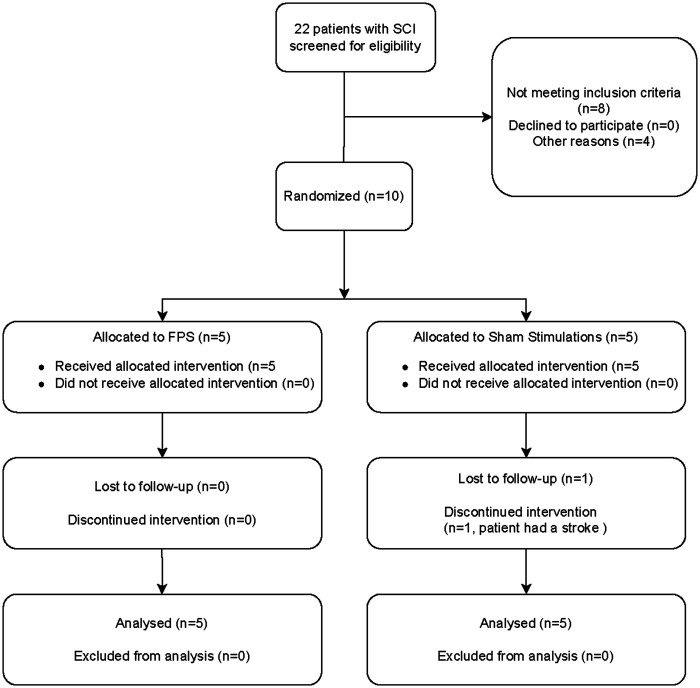
Flowchart representing the inclusion of participants in the clinical trial.

Patients were recruited from March 2022 to March 2024. A total of 22 patients with SCI admitted to ICU were screened; 14 patients met the inclusion criteria, and 10 were ultimately included in the study (Figure 3). One patient was excluded at about three weeks of treatment due to a stroke.

Participant characteristics are summarized in Table 2. The study cohort consisted of ten SCI patients recruited within 10 days post-injury. The sample included four patients with motor complete lesions (three classified as AIS A and one as AIS B) and six with motor incomplete lesions (three classified as AIS C and three as AIS D).

**Table 2:** Participant characteristics.

Subject	NLI	AIS	Intervention	Time from SCI to inclusion (days)	Age (years)	Gender	Height (cm)	Weight (kg)	Cause of SCI	Polytrauma
1	C4	C	FPS	4	53.3	M	175	60	Fall from elevation	Yes
2	C2	D	Sham	4	59.6	M	171	78	Road accident	No
3	C8	A	FPS	1	22.7	M	180	83.5	Assault	Yes
4	C5	C	Sham	8	79.2	M		76.5	Road accident	No
5	C4	A	FPS	3	19.2	M	170	70	Road accident	Yes
6	C3	C	FPS	5	75	F	148	60	Fall from standing height	No
7	C5	D	FPS	10	64.8	M	167	76	Fall from elevation	No
8	T5	A	Sham	2	25.3	F	175	79.5	Fall from elevation	Yes
9	C5	D	Sham	4	61.3	F	175	70	Fall from elevation	No
10	C4	B	Sham	3	63.5	M	187	89	Fall from standing height	No
FPS ^a^		2 (40%)	5 (50%)	4 (2)	53.3 (42.1)	1 (20%)	170 (8)	70 (16)		3 (60%)
Sham ^a^		2 (40%)	5 (50%)	4 (1)	61.3 (3.9)	2 (40%)	175 (4)	78 (3)		1 (20%)
Subject	ISS	IGS2	Urgent spinal surgery	Time from SCI to surgery (hours)	Tracheotomy	Mechanical ventilation duration (days)	Length of ICU stay (days)	Time from injury to start of FPS (days)	Number of sessions	
1	16	17	No	NA	No	0	31	4	31	
2	17	15	Yes	22	No	0		7	11	
3	38	18	Yes	7	Yes	45	50	3	25	
4	16	24	No	NA	No	0	24	8	6	
5	33	8	Yes	8	Yes	43	80	15	21	
6	16	28	Yes	5	No	0	8	5	31	
7	16	18	No	NA	No	0	11	10	8	
8	33	16	Yes	8	No	0	17	9	9	
9	16	28	Yes	6	No	2	23	4	18	
10	25	26	Yes	9	No	1	54	3	28	
FPS ^b^	16 (17)	18 (1)	3 (60%)	7 (1.5)	2 (40%)	0 (43)	31 (39)	5 (6)	25 (10)	
Sham ^b^	17 (9)	24 (10)	4 (80%)	8.5 (4.75)	0 (0%)	0 (1)	23.5 (10)	7 (4)	11 (9)	

NLI, neurological level of injury; AIS, American Spinal Injury Association Impairment Scale; FPS, functional proprioceptive stimulation; Sham, Sham stimulation; ISS, injury severity score; IGS2, simplified acute physiology score II; NA, not applicable.

^a^ Quantitative variables are presented as median (interquartile range). Binary variables are presented as a count (percentage) of “motor complete”, “female” or “yes”, as appropriate. Percentages are calculated within each group.

^b^ Quantitative variables are presented as median (interquartile range). Binary variables are presented as a count (percentage) of “yes”. Percentages are calculated within each group.

All participants, except one, received FPS within 10 days post-injury (median = 4 days for both groups, see Table 2). The exception was a patient who delayed initiation because he wanted to consult his parents. Two patients, both in the FPS group, required the continuation of mechanical ventilation after spinal surgery and subsequently underwent tracheotomy.

Sedation was discontinued after surgery for all patients, with the exception of two who required an additional day of sedation. Noteworthy, no patient received neuromuscular blocking agents (curare) during the study period. No patient received any anti-spasticity medication during the study period, including baclofen or botulinic toxin.

### 3.2 Safety of FPS

#### 3.2.1 Adverse events

No FPS sessions were discontinued based on the criteria for discontinuation or suspension of treatment (see Section 2.6.). Furthermore, no patient reported discomfort or pain during the sessions in either group.

No adverse event directly attributable to the vibration sessions occurred. Specifically, there was no disconnection of catheters, chest tubes, or accidental extubation. The only symptoms observed were slight redness and transient skin depressions around the vibrators, which disappeared between sessions.

The incidence of clinically significant adverse events was similar between the two groups, with the majority being urinary or pulmonary infections. Only one serious adverse event occurred: a patient in the sham stimulation group suffered a cerebral hemorrhage, which occurred some time after a sham stimulation session and was deemed unrelated to the study intervention (Table 3).

**Table 3:** Summary of adverse events by participant and by treatment group.

Subject	Intervention	Pulmonary sepsis	Urinary sepsis	Cerebral hemorrhage	Total
1	FPS	0	1	0	1
2	Sham	0	1	1	2
3	FPS	1	0	0	1
4	Sham	0	0	0	0
5	FPS	2	0	0	2
6	FPS	0	0	0	0
7	FPS	0	0	0	0
8	Sham	0	0	0	0
9	Sham	0	1	0	1
10	Sham	0	1	0	1
**Total by group**	**FPS**	**3**	**1**	**0**	**4**
**Total by group**	**Sham**	**0**	**3**	**1**	**4**

Adverse events were similar in frequency between the FPS and sham groups (*n* = 4 in each group). The majority of events were urinary or pulmonary infections. One case of cerebral hemorrhage was reported in the sham group. Values in bold correspond to totals by group.

#### 3.2.2 No deficit in motor recovery

In both groups, two patients experienced a change in their AIS grade. In the FPS group, one patient improved from AIS A to AIS D, while another progressed from AIS C to AIS D. In the sham stimulation group, one patient improved from AIS A to AIS B, and another progressed from AIS B to AIS D.

**Figure 4:**
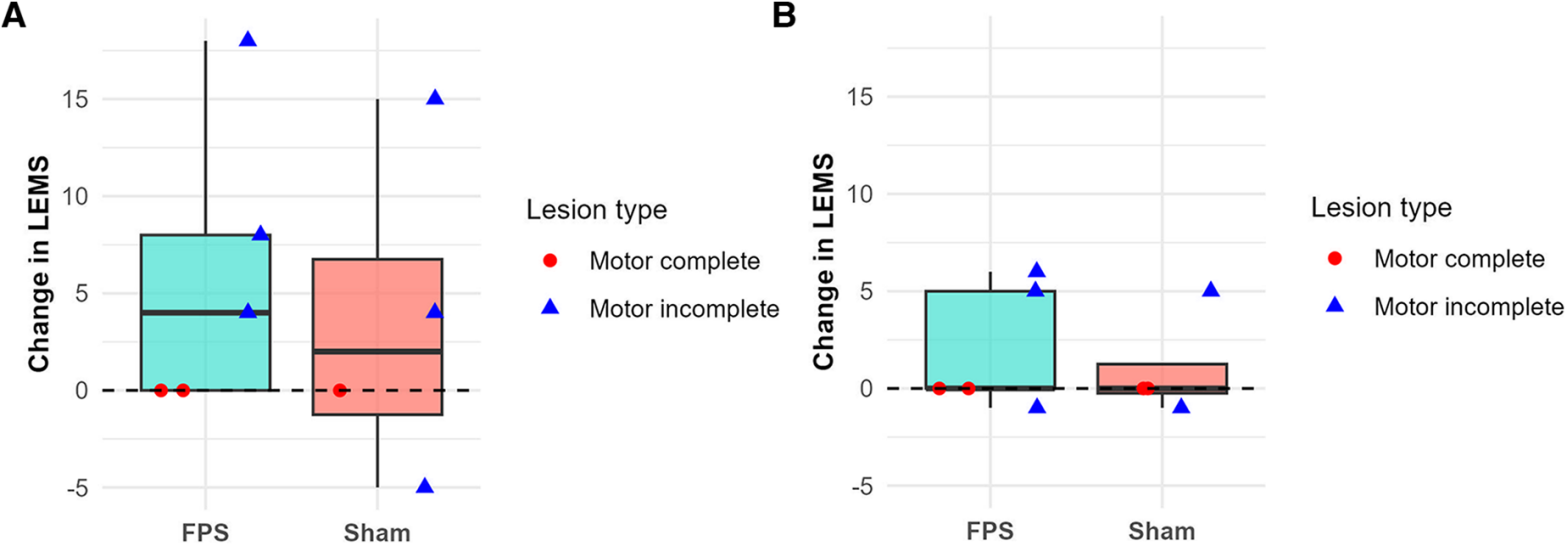
Change in the lower-extremity motor score (LEMS) between the first and second weeks **(A)** and between the second week and the first month **(B)**. Boxplots represent group data (green: FPS group; orange: sham stimulation group). Individual patient data are shown as red-filled circles for motor complete lesions (●) or blue-filled triangles for motor incomplete lesions (▴). LEMS improvements were primarily observed during the first two weeks, particularly in patients with motor incomplete lesions. One motor complete patient in the sham group is missing on panel **(A)**, and one motor incomplete patient in the sham group is missing on panel **(B)**, due to unavailable data.

The evolution of the ISNCSCI motor and sensory scores was comparable between the two groups, particularly regarding the LEMS changes. Figure 4 illustrates the evolution of this score between the first and second weeks, as well as between the second week and the first month.

### 3.3 Effect on spasticity

#### 3.3.1 Time to spasticity onset

**Figure 5:**
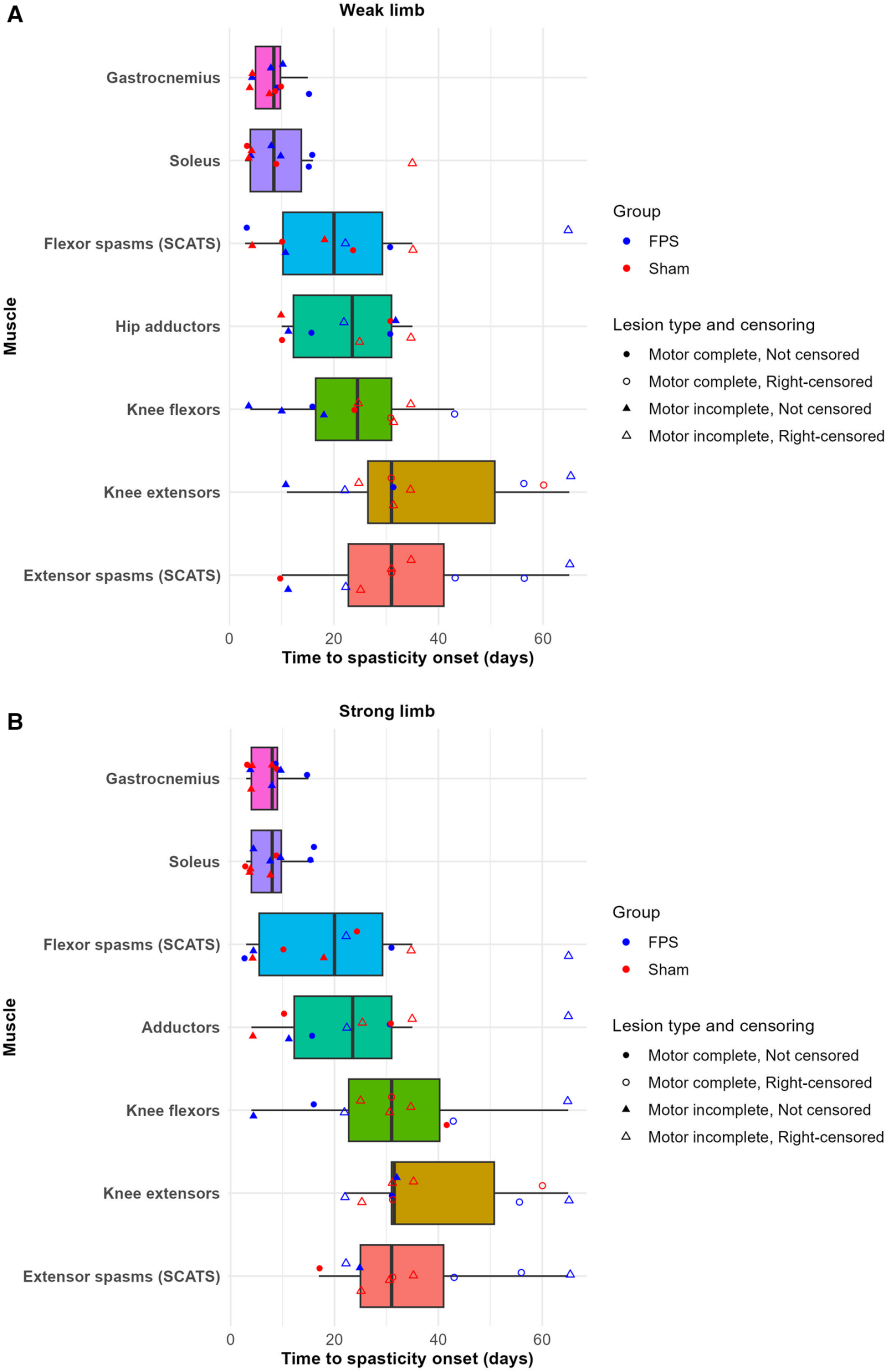
Time to spasticity onset by muscle and group in the weak **(A)** and strong **(B)** lower limbs. Boxplots represent individual muscles. Data points indicate participants: circles (●) denote complete lesions, triangles (▴) denote incomplete lesions. The colors of the points differentiate treatment groups: red for sham stimulation and blue for FPS. For participants who did not develop spasticity in a given muscle during the weekly follow-up period, the last weekly follow-up time was recorded as the onset of spasticity, and these points are indicated as right-censored with unfilled shapes (○ and ▵). Notably, all patients developed spasticity in the gastrocnemius muscle in both limbs, as indicated by the absence of censored data points for this muscle.

No discernible difference was observed between the two groups regarding the time to onset of spasticity by muscle. Figure 5 depicts the interval between SCI and the first examination detecting spasticity in a given muscle. For both groups, irrespective of the lesion type (complete or incomplete), the gastrocnemius and the soleus were the first to exhibit spasticity. By 15 days post-SCI, all patients demonstrated spasticity in the gastrocnemius of both lower limbs and the soleus of the strong lower limb. Only one patient did not exhibit spasticity in the soleus of the weak lower limb at this time point.

Given that the gastrocnemius and soleus consistently exhibited the earliest onset of spasticity, the subsequent analysis focused on these muscles regarding the MAS and the TS.

#### 3.3.2 Medium-term evolution of spasticity

Graphical analysis revealed no discernible difference between the two groups regarding the evolution of the MAS score, TS score, and TS angle for the gastrocnemius and the soleus during the first month post-SCI.

Similarly, no difference was observed in the evolution of ankle flexion amplitude, which reflects the progression of joint stiffness.

Furthermore, no differences were identified in the extensor and flexor spasms components of the SCATS, nor in patients’ self-assessments of spasticity-related discomfort.

**Figure 6:**
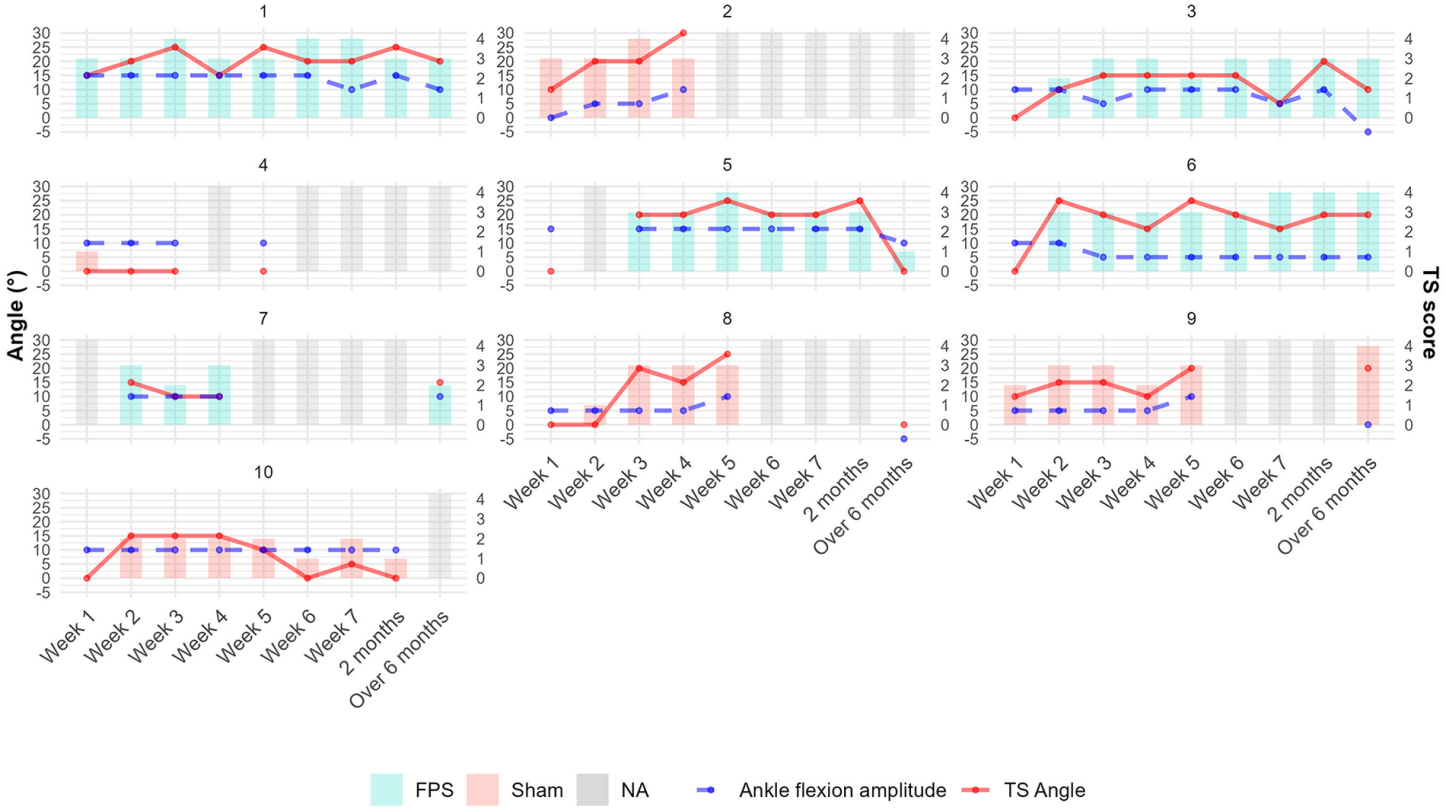
Individual patient trajectories of gastrocnemius spasticity measures and ankle mobility over time in the weak lower limb. Bars represent TS scores. Solid red lines indicate TS angles (degrees); dashed blue lines show ankle flexion amplitudes (degrees). Bar colors denote treatment groups: light red for sham stimulation and turquoise for FPS. Gray bars indicate missing data. TS angle was set to 0 when undefined (TS score of 0 or 1). Patient 3 illustrates a common trend: an increase in spasticity during the first three weeks, followed by a relative plateau and a late decrease in TS angle corresponding with increased ankle stiffness.

Graphical analysis of individual trajectories for these same parameters also did not reveal any group differences. Figure 6 illustrates the individual evolution of spasticity (TS score and TS angle) in the gastrocnemius and ankle flexion amplitude of the weak limb over a maximum period of 6 months. The TS score and TS angle demonstrated rapid progression during the initial weeks, followed by a plateau phase. In contrast, ankle stiffness developed more gradually over time. No significant correlations were found between spasticity measures and lower-limb motor recovery (see Supplementary Material Table S1).

#### 3.3.3 Short-term evolution of spasticity: pre- and post-session comparisons

The graphical analysis of pre- and post-session spasticity measurements of the gastrocnemius and soleus suggests a slight effect of the FPS session on spasticity in both muscles. This effect was observed across multiple measures: the MAS score, the TS degree of resistance, the TS angle, and the ankle flexion amplitude.

**Figure 7:**
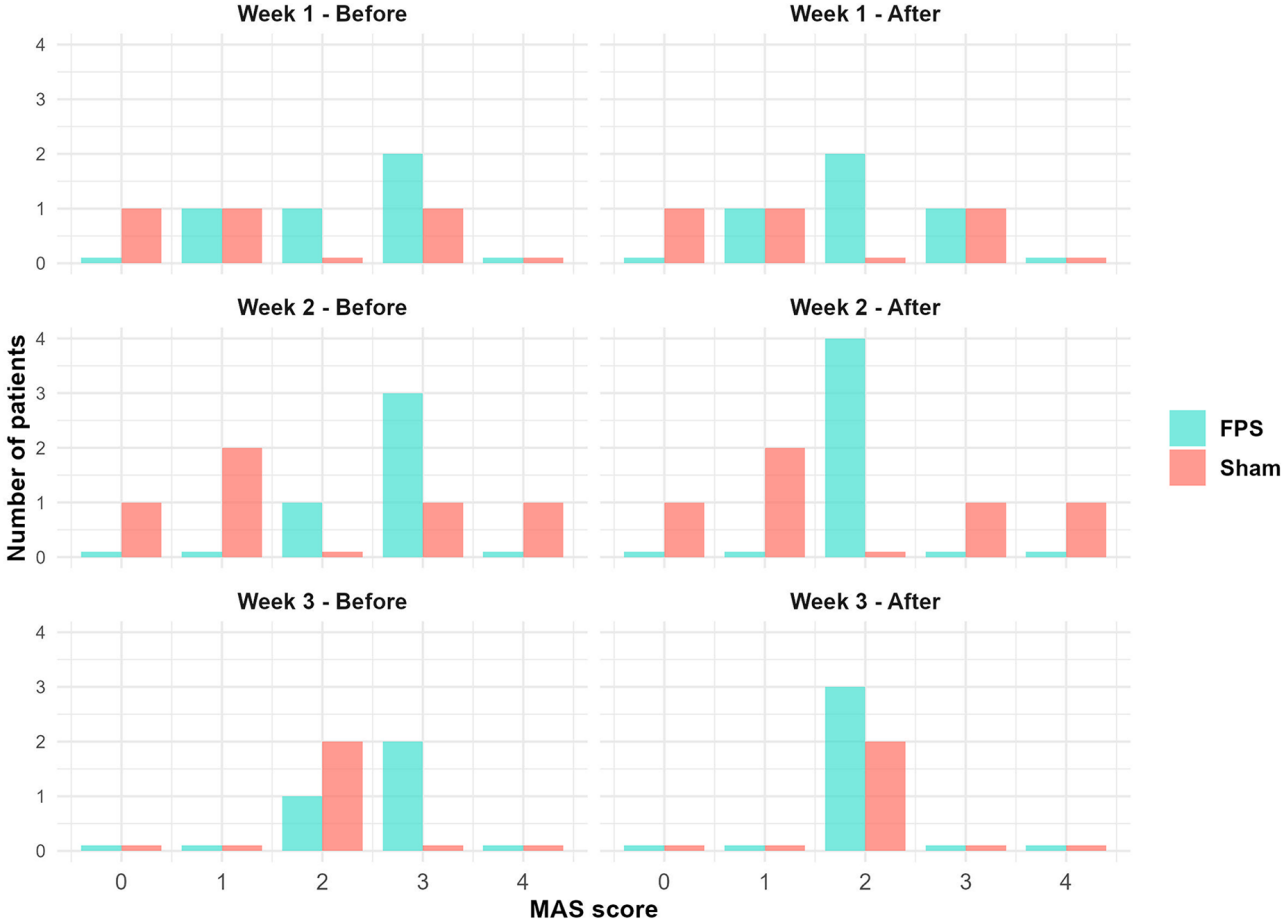
Distribution of participants by MAS scores for gastrocnemius spasticity in the weak lower limb: Pre- and post-vibration session comparisons by treatment group. Bar colors denote treatment groups: light red for sham stimulation and turquoise for FPS. For each MAS score, bars representing each group are displayed side-by-side. The FPS group shows a trend towards lower MAS scores post-session, while changes in the sham group appear less pronounced.

Figure 7 illustrates this effect by showing the repartition of patients according to the MAS score of the gastrocnemius of the weak lower limb. In the sham stimulation group, rare changes were observed from pre- to post-vibration sessions. Changes were more pronounced in the FPS group. However, these differences did not reach statistical significance (see Supplementary Material Figure S1).

### 3.4 Effect on muscle atrophy

#### 3.4.1 Muscle atrophy rates during the first 45 days

**Figure 8:**
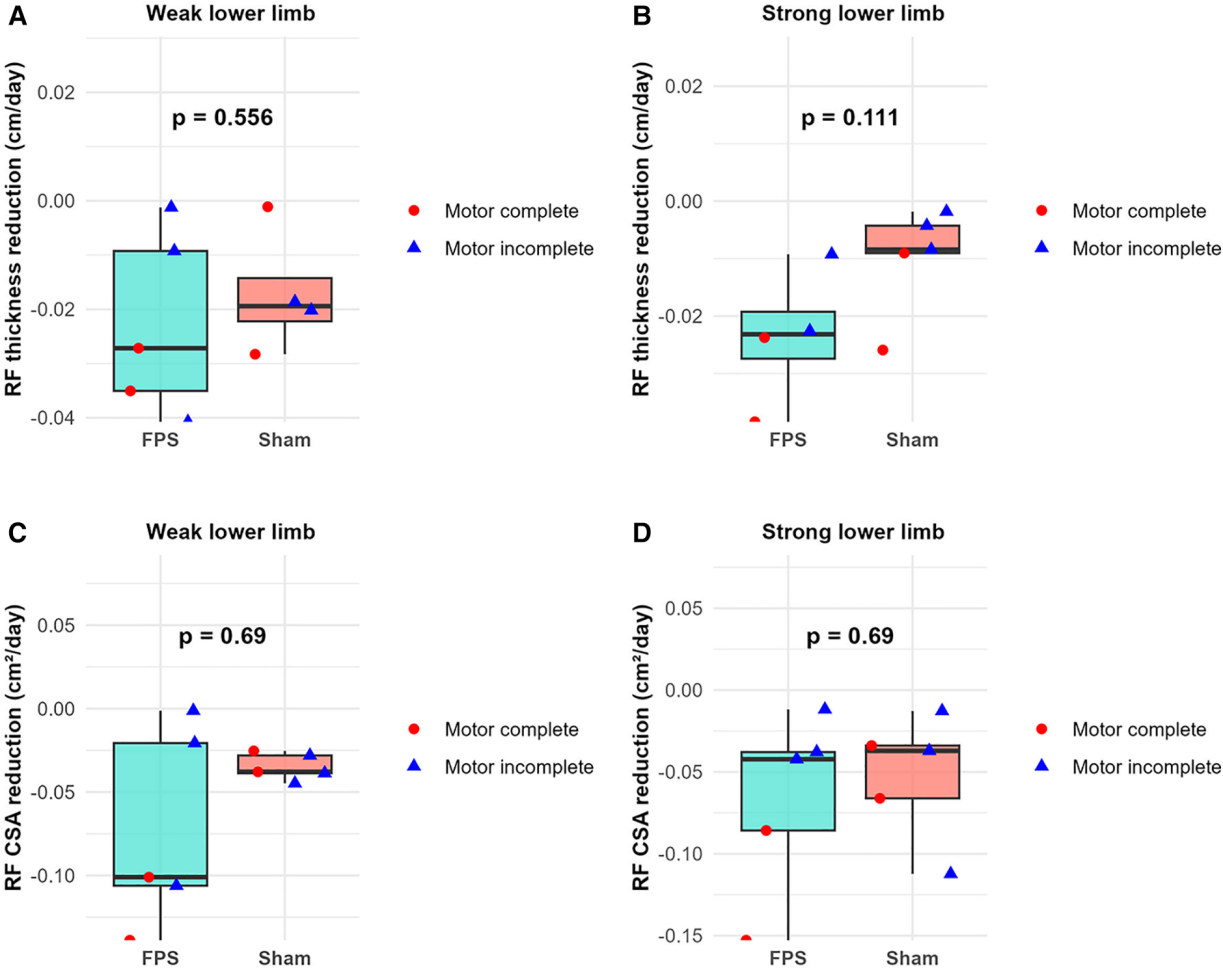
Between-group comparison of rectus femoris (RF) atrophy rates during the first 45 days post-injury. **(A,B)** RF thickness reduction rate (cm/day) for weak **(A)** and strong **(B)** lower limbs. **(C,D)** RF cross-sectional area (CSA) reduction rate (cm²/day) for weak **(C)** and strong **(D)** lower limbs. Boxplots represent group data (green: FPS group; orange: sham stimulation group). Individual patient data are shown as red-filled circles for motor complete lesions (●) or blue-filled triangles for motor incomplete lesions (▴).

No statistically significant difference was observed between the two treatment groups regarding the slope of the rectus femoris thickness reduction and the cross-sectional area reduction during the initial 45-day period. Graphical analysis of the data corroborates this finding (Figure 8).

**Figure 9:**
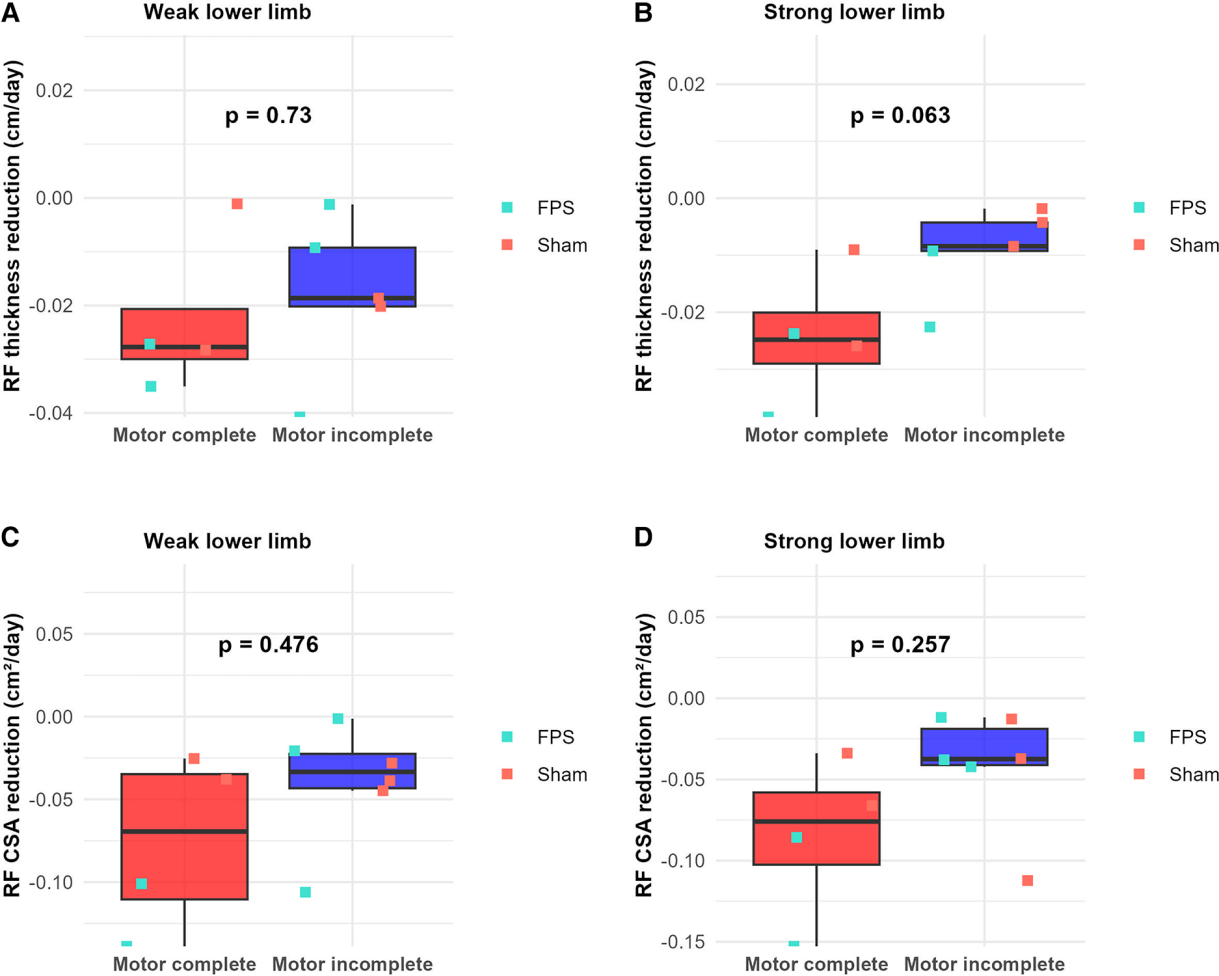
Comparison of rectus femoris (RF) atrophy rates between motor complete and motor incomplete lesions during the first 45 days post-injury. **(A,B)** RF thickness reduction rate (cm/day) for weak **(A)** and strong **(B)** lower limbs. **(C,D)** RF cross-sectional area (CSA) reduction rate (cm²/day) for weak **(C)** and strong **(D)** lower limbs. Boxplots represent data of motor complete and motor incomplete lesions (red: complete motor lesions; blue: incomplete motor lesions). Individual patient data are shown as green-filled squares for the FPS group (▪) or orange-filled squares for the sham stimulation group (▪).

Conversely, graphical analysis suggests a potential distinction between motor complete and motor incomplete participants regarding the slope of rectus femoris thickness reduction and the cross-sectional area reduction over the same 45-day interval. The slope appears steeper for motor complete participants (Figure 9). However, subsequent statistical analysis failed to reveal any significant parameter differences between these two subgroups.

## 4 Discussion

This pilot study assessed the safety, feasibility, and potential effects of early functional proprioceptive stimulation (FPS) in patients suffering from spinal cord injury (SCI). Results revealed no differences in the occurrence of adverse events between the groups receiving early FPS and sham stimulation. Furthermore, no adverse events were associated with the use of FPS, and no sessions had to be discontinued because of patient instability or discomfort. These findings indicate that early FPS is feasible and can be administered safely as soon as the patient is in the intensive care unit (ICU). This aligns with Dionne et al. preliminary results on the safety of passive cycling implemented as early as 24 h post-decompressive surgery in traumatic SCI patients (43). Our results do not allow us to draw conclusions about the medium- or long-term effects of early FPS on muscle spasticity and atrophy. However, a more in-depth analysis at the session level indicates a transient effect of FPS on spasticity. This observation corroborates previous findings for focal vibration (17, 21).

A notable finding of this study is the short delay in spasticity onset, particularly in the gastrocnemius and soleus muscles. These muscle groups may serve as effective sentinels for monitoring early spasticity in SCI patients. We initially hypothesized that early FPS could delay spasticity onset. However, several patients already exhibited signs of spasticity when treatment began. And all patients presented gastrocnemius spasticity on both lower limbs and in the soleus of the strong lower limb 15 days after SCI. Since spasticity was first evaluated within the first 10 days and then assessed weekly, the onset of spasticity is determined with an uncertainty of about one week. This observation is crucial as gastrocnemius and soleus spasticity leads to ankle stiffness and equinus deformity (44). Our findings are consistent with Richard-Denis et al.'s longitudinal study on early spasticity after SCI, which reported that all patients who developed spasticity exhibited signs within the first month post-injury. However, while we had a 100% prevalence of early spasticity, the literature reports prevalences ranging from 52% to 89% in the first post-traumatic weeks (11, 13, 45–47). This discrepancy may be attributed to the inherent subjectivity of clinical spasticity assessment (30). A key strength of our study is that a single examiner assessed spasticity for all patients across all time points. This consistency allowed for the detection of subtle changes in spasticity tests, potentially capturing milder cases that might have been overlooked in studies with multiple examiners. This approach may have contributed to our higher reported prevalence but also ensured a more uniform and sensitive assessment of spasticity progression over time.

Our results show a tendency toward a beneficial short-term effect of FPS on spasticity, accompanied by subjective sensations of muscular relaxation in some patients, although not quantified in our results. Even if FPS does not have long-term effects on spasticity, it could be valuable in temporarily alleviating spasticity-induced stiffness. This could facilitate work on muscle flexibility and, where appropriate, strengthening antagonist muscles following a FPS session. Importantly, this approach could be implemented early in the ICU stay.

The hypothesized mechanisms of FPS action on spasticity may be similar to those of transcutaneous and epidural spinal cord stimulations (48, 49), as these techniques are all assumed to stimulate proprioceptive afferents. Potential mechanisms include the restoration of chloride homeostasis and, subsequently, of postsynaptic inhibition, the restoration of homosynaptic depression (49), and, more generally, the normalization of neural circuit excitability (50). Compared to other sensory stimulation techniques, FPS offers the advantage of being non-invasive and simple to administer, making it particularly suitable for early intervention in ICU settings.

Another benefit of early FPS is that it stimulates patients and prompts them to request additional sessions of stimulation. This aspect is significant, particularly for high SCI patients who often experience prolonged ICU stays, sometimes with complications and limited rehabilitation options. Most patients, including those in the sham stimulation group, expressed satisfaction and desire for stimulation sessions, thus indicating a demand for more rehabilitation at this early stage. Even in the sham stimulation group, the avatar on the screen likely encouraged motor imagery and contraction efforts. One patient in the sham stimulation group referred to it as “her little coach”. This positive engagement suggests that early rehabilitation interventions may have psychological benefits beyond their physical effects, an aspect that deserves to be formally evaluated in future studies.

Our pilot study presents several strengths and limitations. Therefore, it offers valuable insights for future research. The diversity of injury severities and levels provides a representative sample for evaluating early FPS in acute SCI patients. However, the small sample size limits our ability to assess the effects of early FPS with sufficient statistical power. Moreover, motor completeness of the lesion emerged as a decisive factor in evaluating muscle atrophy effects. This is consistent with a previous study that used quantitative computed tomography to measure muscle cross-sectional area and density (51). Although we initially planned to analyze patients with complete and incomplete motor lesions separately, the limited number of patients prevented us from adopting this approach. Therefore, the small sample size not only limited our statistical power but also prevented us from exploring potential differences in FPS efficacy based on injury characteristics. Larger studies are needed to identify the patients who could benefit most from this intervention.

Another critical aspect to consider is the potential dose-dependent efficacy of the treatment. Frequency of administration and total duration may both play a significant role. This relationship has been observed in other therapeutic interventions (52, 53). One patient (patient 4) received only six FPS sessions, likely insufficient to produce a medium or long-term effect. Even if early treatment is feasible, we can assume that insufficient dosage may limit effectiveness. Therefore, it is important to ensure continuity in the patient's rehabilitation pathway. Our initial protocol aimed to continue vibration treatment for eight weeks across different rehabilitation structures, but organizational constraints prevented the completion of the entire therapeutic procedure. We recommend that future studies anticipate and address this issue.

Our study also highlights limitations in spasticity assessment tools. Spasticity scales may lack sensitivity to reflect early-stage spasticity evolution. After an initial increase, spasticity appeared to rapidly reach a plateau lasting at least a month. This is in contradiction with our clinical experience. The Modified Ashworth Scale (MAS), in particular, has demonstrated already documented limitations (54). For instance, in one patient (patient 8), the weak limb's gastrocnemius MAS score returned to 0 after reaching 4, while passive amplitude decreased from 10° to −5° between the last two examinations. This suggests that muscle stiffness may mask spasticity in some cases. Additionally, in some patients, gastrocnemius spasticity disappeared, presumably due to motor recovery [weak limb of patient 4 (sham group), strong limb of patient 7 (FPS group)], thus complicating spasticity analysis during recovery. Additional measurements could provide valuable insights into these cases and the effects of FPS on spasticity. For instance, shear wave elastography could offer valuable insights into the impact of FPS on musculotendinous stiffness. Recent research by Chen et al. (55) demonstrated that focal vibration can enhance the viscoelastic properties of targeted muscles. This may be one of the mechanisms involved in the short-term effect of FPS. Surface electromyography (sEMG) could also be a valuable tool. This method could be used to examine the effects of FPS on spontaneous muscle activity at rest or during passive manoeuvres (56).

In line with the aim to refine assessments. sEMG could also help quantify both acute and chronic effects of FPS on voluntary muscle activation during and after the intervention program (56, 57). Other techniques, such as electroencephalography (EEG) (58) or functional near-infrared spectroscopy (fNIRS) (59), could also help verify if a cortical signature of the FPS is present for SCI patients, in particular those with complete motor lesions.

Research on acute-stage SCI rehabilitation remains complex due to the lack of standardization of interventions and the overlapping of spontaneous recovery (60). Evidence favoring specific interventions or the optimal time to start rehabilitation interventions following SCI is lacking (7, 61), with population heterogeneity posing an additional challenge (62). However, recent evidence confirms that ICU patients can be mobilized early, even with vasopressor treatment (63). Animal studies have demonstrated the potential benefits of early rehabilitation after SCI (64, 65), with one suggesting that responsiveness to rehabilitation diminishes when its initiation is delayed (65). Hence, we advocate for initiating rehabilitation of patients with SCI as soon as they are deemed medically stable.

To implement such an early rehabilitation, the importance of specialized care centers for SCI patients is also emphasized in existing guidelines (6, 66). This study aligns with these efforts toward an optimal care organization for SCI patients. We demonstrated the safety and feasibility of early FPS in acute SCI patients, positioning it as a potential component in early SCI management. Our study also highlights important considerations for future research, including the need for larger, stratified samples and protocols to document early FPS effect and adequate treatment dosage across the rehabilitation continuum.

## Data Availability

The raw data supporting the conclusions of this article will be made available by the authors, without undue reservation.
