# Supplementary material for: Early functional proprioceptive stimulation in high spinal cord injury: a pilot study
Source: Front Rehabil Sci. 2025 Feb 26;6:1490904. doi: 10.3389/fresc.2025.1490904 (PMC11897281; doi:10.3389/fresc.2025.1490904)
Supplement: Supplementary file 1 [file Table1.docx]

# **Supplementary material**

Table S1. Spearman’s Correlation Coefficients between changes in the Lower-Extremity Motor Score (LEMS) and spasticity.

|  | **MAS Score weak** | **MAS Score strong** | **TS Score weak** | **TS Score strong** | **SCATS total weak** | **SCATS total strong** |
| --- | --- | --- | --- | --- | --- | --- |
| LEMS of the weak lower limb | 0.02 | -0.23 | 0.18 | 0.01 | 0.15 | -0.08 |
| LEMS of the strong lower limb | 0.06 | 0.01 | 0.28 | 0.14 | 0.14 | 0.07 |
| *MAS Score weak: MAS score of the gastrocnemius of the weak lower limb; MAS Score strong: MAS score of the gastrocnemius of the strong lower limb; TS Score weak: TS degree of resistance of the gastrocnemius of the weak lower limb; TS Score strong: TS degree of resistance of the gastrocnemius of the strong lower limb; SCATS total weak: Total SCATS score of the weak lower limb; SCATS total strong: Total SCATS score of the strong lower limb.* | | | | | | |

Figure S1. Changes in spasticity scores (A) and spasticity angles (B) from pre- to post-session by treatment group.

Gas: gastrocnemius; Sol: soleus; SL: strong limb; WL: weak limb. Amplitude Gas and Amplitude Sol designate, respectively, the ankle dorsiflexion with the knee extended and with the knee flexed.

Boxplots represent group data (green: FPS group; orange: sham stimulation group).

The p-values correspond to the test for differences in changes pre- and post-session between the two treatment groups. No p-value corresponds to a statistically significant difference (p < 0.05).
