# Supplementary figures and images for: Early functional proprioceptive stimulation in high spinal cord injury: a pilot study
Source: Front Rehabil Sci. 2025 Feb 26;6:1490904. doi: 10.3389/fresc.2025.1490904 (PMC11897281; doi:10.3389/fresc.2025.1490904)

# Supplementary Figures S1 & S2.

Panel A

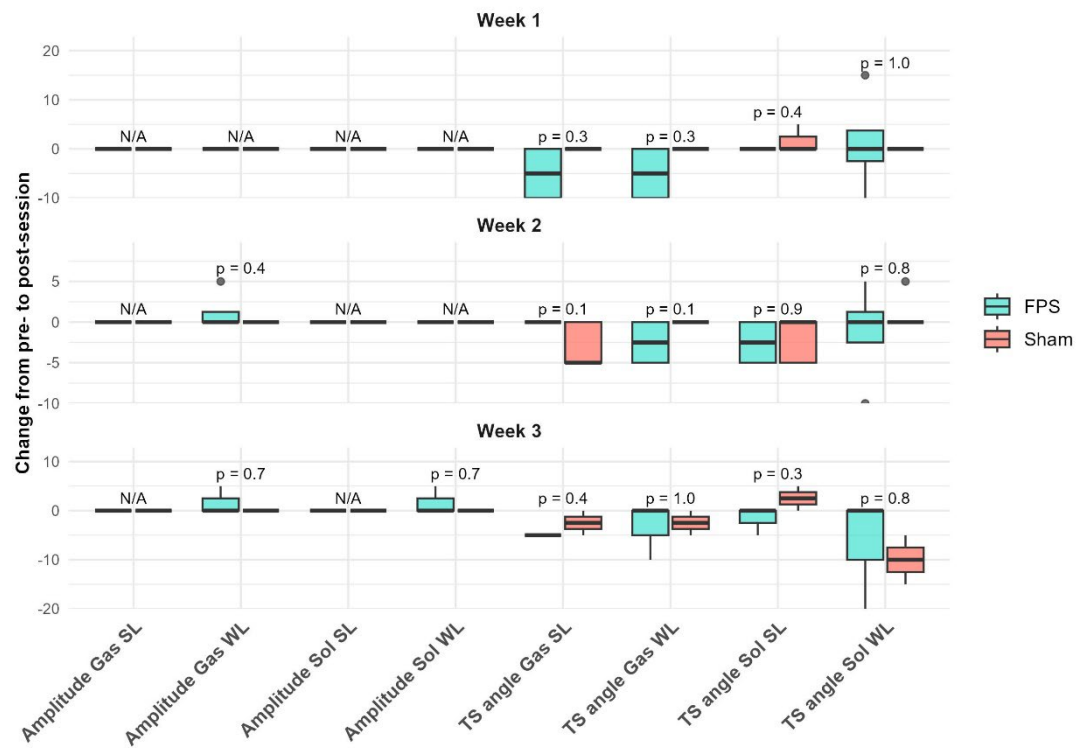

Panel B

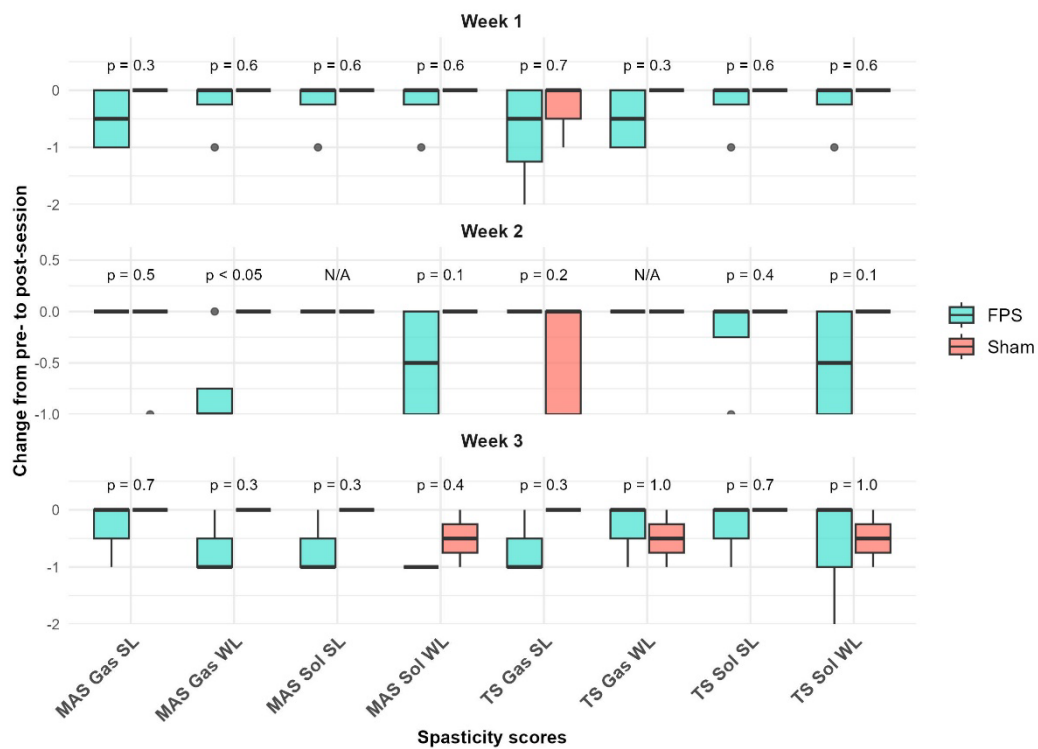

Supplement: Supplementary file 2 [file Image1.pdf]
